# Effects of Additional Yoga Intervention in Children and Adolescents with Major Depressive Disorder: A Randomized Controlled Trial

**DOI:** 10.1192/j.eurpsy.2025.380

**Published:** 2025-08-26

**Authors:** B. N. Patra, R. Sagar, G. Sharma

**Affiliations:** 1 Psychiatry; 2 Cardiology, AIIMS, NEW DELHI, New Delhi, India

## Abstract

**Introduction:**

Depression is one of the most common mental health disorders in children and adolescents. In India, many parents resist psychotropic medication for children due to potential side effects, highlighting the need for non-pharmacological interventions like yoga.

**Objectives:**

The current study investigates the impact of additional yoga therapy on depressive symptoms, global functioning, and parental stress among children and adolescents diagnosed with Major Depressive Disorder (MDD) on children seeking treatment at the Department of Psychiatry of a tertiary care medical institute in India.

**Methods:**

This study included 80 participants aged 6 to 17 years. After taking written informed consent from the parents and assents from the adolescents, they were randomized into two groups: one receiving yoga Therapy alongside treatment as usual (TAU) and the other a waitlist control group receiving only TAU. Assessments were done on both children and their parents, and the instruments included were Centre for Epidemiological Studies Depression Scale for Children (CES-DC), the Children’s Global Assessment Scale (CGAS), the Clinical Global Impression scale (CGI), and the Depression Anxiety and Stress Scale (DASS) for parents. Follow-up assessments occurred at 6 and 12 weeks.

**Results:**

In the experimental group, CES-DC scores showed significant improvements, with p value < 0.01 at 6 weeks and < 0.01 at 12 weeks. Global functioning scores also improved, recording p values of < 0.01 at 6 weeks and < 0.01 at 12 weeks. The control group also exhibited results, with CES-DC p values of < 0.01 at 6 weeks and < 0.01 at 12 weeks. Global functioning scores revealed p values of < 0.01 at 6 weeks and < 0.01 at 12 weeks. However, there were no significant differences in the improvement in CES-DC score and functioning in the experimental and control group at the end of 6 weeks and 12 weeks. At the baseline, at end of 6 weeks and at the end of 12 weeks, there were no significant differences in parental depression, anxiety, and stress score.

**Conclusions:**

Yoga therapy was beneficial for the children and adolescents with major depressive disorder. However, there were no significant differences in the improvement in depression and functioning in the experimental and control group.

**Disclosure of Interest:**

B. Patra Grant / Research support from: The Department of Science & Technology, Govt of India under its scheme “SATYAM”, R. Sagar: None Declared, G. Sharma: None Declared

